# The Stimulation of IGF-1R Expression by Lewis(y) Antigen Provides a Powerful Development Mechanism of Epithelial Ovarian Carcinoma

**DOI:** 10.3390/ijms12106781

**Published:** 2011-10-14

**Authors:** Dawo Liu, Juanjuan Liu, Changzhi Wang, Bei Lin, Qing Liu, Yingying Hao, Shulan Zhang, Masao Iwamori

**Affiliations:** 1 Department of Obstetrics and Gynecology, Shengjing Hospital Affiliated to China Medical University, Shenyang 110004, China; E-Mails: cyldw2007@163.com (D.L.); juanjuanliu_lg@yahoo.com.cn (J.L.); qingLiu@126.com (Q.L.); liushuicc@sina.com (Y.H.); zhangshl99@126.com (S.Z.); 2 Department of Obstetrics and Gynecology, The Second Affiliated Hospital of Dalian Medical University, Dalian 116027, China; E-Mail: changzhix_wang@yeah.net; 3 Department of Biochemistry, Faculty of Science and Technology, Kinki University, 3-4-1 Kowakae, Higashiosaka, Osaka 577-8502, Japan; E-Mail: iwamori@163.com

**Keywords:** epithelial ovarian tumor, Insulin-like growth factor receptor-1, Lewis(y) antigen, immunohistochemistry, immunofluorescence double labeling method

## Abstract

**Objective:**

This study aimed to measure and correlate the expression of insulin-like growth factor receptor-1 (IGF-1R) and the Lewis(y) antigen in ovarian cancer cell lines and tissue samples.

**Methods:**

Reverse transcriptase PCR (RT-PCR), Western blotting, immunoprecipitation, immunohistochemistry, and immunofluorescence double-labeling techniques were applied to detect and measure the expression of Lewis(y) and IGF-1R.

**Results:**

In α1,2-fucosyltransferase (α1,2-FT)-transfected cells, IGF-1R expression was significantly upregulated compared with cells that do not overexpress α1,2-FT (*P* < 0.05). The amount of Lewis(y) expressed on IGF-1R increased 1.81-fold in α1,2-FT-overexpressing cells (*P* < 0.05), but the ratio of Lewis(y) expressed on IGF-1R to total IGF-1R was unaltered between two cells (*P* > 0.05). In malignant epithelial ovarian tumors, the positivity rates of Lewis(y) and IGF-1R detection were 88.3% and 93.33%, respectively, which is higher than the positivity rates in marginal (60.00% and 63.33%, all *P* < 0.05), benign (33.00% and 53.33%, all *P* < 0.01), and normal (0% and 40%, all *P* < 0.01) ovarian samples. No correlations were detected in positivity rates of Lewis(y) or IGF-1R expression with respect to clinicopathological parameters in ovarian cancers (all *P* > 0.05). Both IGF-1R and Lewis(y) were highly expressed in ovarian cancer tissues, and their expression levels were positively correlated (*P* < 0.05).

**Conclusion:**

Overexpression of Lewis(y) results in overexpression of IGF-1R. Both IGF-1R and Lewis(y) are associated with the occurrence and development of ovarian cancers.

## 1. Introduction

Ovarian cancer has the highest mortality of all female genital tract tumors, and effective early diagnostic methods are lacking. Alterations in cell membrane-associated carbohydrate antigens are closely linked with malignancy, invasion, and metastasis of cells. Ovarian cancer is characterized by changes in type II sugar chains, and more than 75% of epithelial ovarian cancers overexpress the Lewis(y) antigen to some extent [1]. Lewis(y) antigen is a difucosylated oligosaccharide containing two fucoses, and is carried by glycoconjugates (glycoproteins and glycolipids) on the plasma membrane. Lewis(y) antigen is expressed predominately during embryogenesis. Under normal physiological conditions, its expression in adults is restricted to the surface of granulocytes and epithelium [2]. However, overexpression of Lewis(y) antigen is frequently found in human cancers and has been shown to be associated with poor prognosis [3,4].

We previously transfected the ovarian cancer cell line RMG-I with human α1,2-fucosyltransferase (α1,2-FT) gene, which catalyzes the fucosylation of Lewis(y), to establish the Lewis(y)-overexpressing cell line, RMG-I-H. Compared to RMG-I cells, which express low amounts of Lewis(y) antigen, RMG-I-H cells exhibit enhanced proliferation, adhesion, invasion, metastasis, and drug resistance, indicating that Lewis(y) plays a critical role in the progression to and resistance of ovarian cancer [5–7].

Insulin-like growth factor receptor-1 (IGF-1R) is a transmembrane glycoprotein consisting of two α, β chains. Among the human IGF system reported to date, IGF-1R plays a vital role in fetal growth and linear growth of the skeleton and other organs [8]. High level expression of IGF-1R has been implicated in several different cancers. Abundant data from cell culture, animal, and human epidemiologic studies have suggested that IGF-IR regulate all aspects of the malignant phenotype [9–12] according to its extent of glycosylation [13].

In the present study, we investigated the expression and the correlation of Lewis(y) and IGF-1R in ovarian serous and mucinous carcinoma tissue specimens by using immunohistochemical method. At the same time, double-labeling immunofluorescence, co-immunoprecipitation, Western blotting and PCR methods were used to further elucidate the correlation of Lewis(y) antigen and IGF-1R. Our study provides a theoretical mechanism of ovarian carcinogenesis, development mechanisms, and a possible target for the development of biological treatment.

## 2. Results

### 2.1. IGF-1R mRNA and Protein Expression in RMG-I and RMG-I-H Cells before and after Antibody Blocking

**Figure 1:**
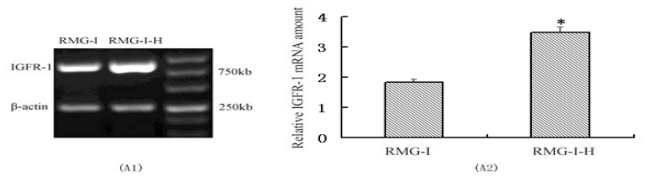

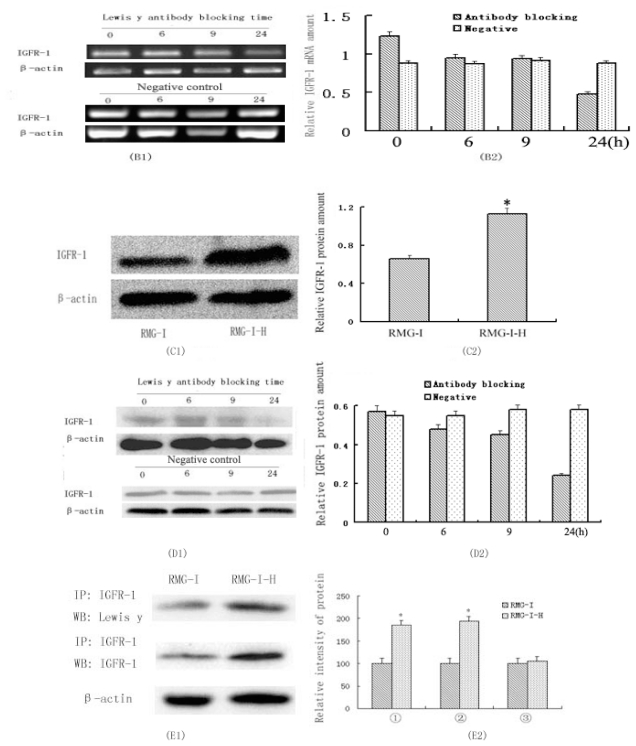
Analysis of IGF-1R after cells transfected with α1,2-FT gene and the expression of IGF-1R of RMG-I-H cell line in control and anti-Lewis(y) monoclonal antibody treated groups (A, B, C, D), and expression of IGF-1R proteins and the Lewis(y) content of the glycans of IGF-1R before and after α1,2-FT gene transfection (E). A1: RT-PCR profiles of IGF-1R in RMG-I and RMG-I-H cell lines. B1: RT-PCR results of mRNA expression of IGF-1R of RMG-I-H cell line in control and anti-Lewis(y) monoclonal antibody treated groups. C1: Western Blot profiles of IGF-1R in RMG-I and RMG-I-H cell lines. D1: Western Blot results of protein expression of IGF-1R of RMG-I-H cell line in control and anti-Lewis(y) monoclonal antibody treated groups. E1: Western blot profiles of immunoprecipitated IGF-1R protein using corresponding antibodies and Lewis(y) antibody. A2, B2, C2, and D2: Relative amount of RT-PCR or Western Blot profiles. E2: Densitometric quantification of IGF-1R ➀ and Lewis(y), ➁ in E1 and calculation of Lewis(y) expression/IGF-1R ➂ (set the RMG-I cells as 100%) (*n* = 3). ^*^ *P* < 0.01 compared to RMG-I. (IP: Immunoprecipitation by the antibody to IGF-1R; WB: Western immunoblot with the antibodies to IGF-1Ror Lewis(y)). A-E are the representative of three independent and reproducible experiments.

The relative expression of IGF-1R mRNA (3.49 ± 0.08) in RMG-I-H cells, as measured by RT-PCR, was significantly higher than that in RMG-I cells (1.84 ± 0.04) (*P* < 0.05) [Figure 1(A)]. After Lewis(y) antigen on RMG-I-H cells was blocked by 10 μg/ml of monoclonal anti-Lewis(y), the expression of IGF-1R mRNA decreased gradually with duration of blocking (*P* < 0.05), reaching a minimum at 24 h of blocking [Figure 1(B)]. Similarly, Western blotting demonstrated that the relative expression of IGF-1R protein (1.13 ± 0.07) in RMG-I-H cells was significantly higher than that in RMG-I cells (0.66 ± 0.12) (*P* < 0.05) [Figure 1(C)], and when Lewis(y) was blocked with monoclonal antibody, the expression of IGF-1R protein decreased gradually with treatment time (*P* < 0.05), reaching a minimum at 24 h [Figure 1(D)].

### 2.2. Expression of IGF-1R Protein and Lewis(y) on the Surface of RMG-I and RMG-I-H Cells and in Epithelial Ovarian Cancer Tissues

**Figure 2:**
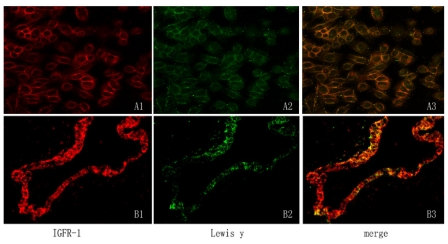
IGF-1R and Lewis(y) colocalize in ovarian carcinomar cell RMG-I-H and ovarian malignant tumor; using double-labeling immunofluorescence method. IGF-1R (A1, B1), Lewis(y) (A2, B2), merged image (A3, B3, original magnification × 400).

The expression of Lewis(y) on IGF-1R was observed by immunoprecipitation of IGF-1R and Western blotting with a monoclonal antibody against Lewis(y). The total amount of Lewis(y) on IGF-1R was increased in α1,2-FT-transfected RMG-I-H cells by up to 1.81-fold compared to RMG-I cells (*P* < 0.05). However, the ratio of total Lewis(y) on IGF-1R to total IGF-1R protein was unaltered because total IGF-1R was elevated to the same magnitude as Lewis(y) (*P* > 0.05) [Figure 1(E)]. Immunofluorescence double-labeling of RMG-I-H cell and ovarian cancer tissue detected Lewis(y) predominantly localized to the cell membrane (green fluorescence) and IGF-1R localizing mostly to the cell membrane but occasionally to the cytoplasm (red fluorescence). Merged images suggested colocalization of IGF-1R and Lewis(y) on cytolemma (Figure 2, yellow fluorescence).

### 2.3. Expression of Lewis(y) and IGF-1R in Various Ovarian Tissues

In malignant epithelial ovarian tumors, Lewis(y) expression was generally increased, with staining localizing primarily to the cell membrane and occasionally to the cytoplasm. The positivity rate of Lewis(y) detection in these samples was 88.33%, substantially higher than the positivity rate in marginal samples (60.00%) (*P* < 0.05) and in benign tumors (33.33%) (*P* < 0.01). The difference in positivity rates of Lewis(y) between marginal ovarian tumors and benign tumors was not statistically significant (*P* > 0.05). In normal epithelial ovarian tissues, the Lewis(y) antigen was not detected (Table 1).

**Figure 3:**
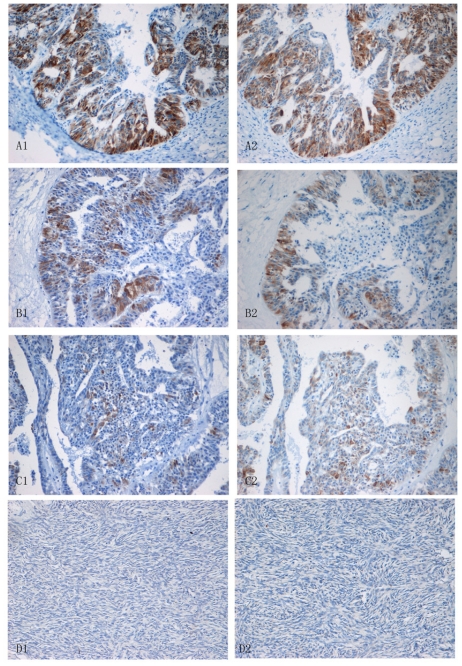
Immunohistochemical staining in ovarian malignant tumor (A1, A2), borderline tumor (B1, B2), benign tumor (C1, C2), and normal ovarian tissue (D1, D2). IGF-1R (A1, B1, C1, D1) and Lewis(y) (A2, B2, C2, D2; original magnification × 200).

The expression of IGF-1R was mainly localized to the cell membrane with sparse localization to the cytoplasm and nucleus. In malignant epithelial ovarian tumors, the positive rate of IGF-1R detection (93.33%) was significantly higher than those in marginal (63.33%) (*P* < 0.05), benign (53.33%) (*P* < 0.05), and normal ovarian samples (40%) (*P* < 0.01). Pairwise comparisons between marginal, benign, and normal ovarian samples identified no significant differences in positivity rates (all *P* > 0.05) (Table 1, Figure 3).

### 2.4. Relationship between Expression of IGF-1R and Antigen Lewis(y), and Clinical Features of Ovarian Cancer

In ovarian serous cystadenoma tissues, the positivity rate of Lewis(y) detection was 90.00% and did not differ significantly from the positivity rate in mucinous cystadenoma (86.67%) (*P* > 0.05). The positivity rate of Lewis(y) detection in stage III–IV ovarian cancer was 95.24%, which was not significantly higher than that in stage I–II (84.62%) (*P* > 0.05). The positivity rates of Lewis(y) among high-, moderate- and low-differentiation groups of ovarian cancer were 80.95%, 85.71%, and 100%, respectively, but this increase in positivity rate with a decrease in differentiation level was not statistically significant (*P* > 0.05). The positivity rate of Lewis(y) detection in the lymph node metastasis group (100%) was not significantly higher than that in the lymph node metastasis-free group (85.42%) (*P* > 0.05) (Table 2).

The positivity rates of IGF-1R detection in serous and mucinous cystadenoma were 90% and 80%, respectively, which did not represent a significant difference (*P* > 0.05). The positivity rates of IGF-1R in ovarian cancer stages III–IV and I–II were 85.71% and 84.62%, respectively, which also was not significant (*P* > 0.05). In high-, moderate-, and low-differentiation ovarian cancer tissues, the positivity rates of IGF-1R were 76.19%, 85.71%, and 94.44%, respectively, and no statistical significance was detected among the three group comparisons (*P* > 0.05). The positivity rate of IGF-1R in the lymph node metastasis group (91.67%) was not significantly higher than that in the lymph node metastasis-free group (93.33%) (*P* > 0.05) (Table 2).

### 2.5. Staining Intensity of Lewis(y) and IGF-1R with Relation to Clinicopathological Parameters of Ovarian Cancer

We detected and analyzed the staining intensity of ovarian cancer sections that were positive for Lewis(y) by immunohistochemistry. In stage III–IV ovarian cancer, the mean optical density of Lewis(y) was 0.505 ± 0.072, which was significantly higher than the stage I–II value of 0.455 ± 0.065 (*P* < 0.05). In low-differentiation ovarian cancer tissue positive for Lewis(y), the mean optical density of Lewis(y) was 0.498 ± 0.084, which is significantly higher than the value obtained for high-differentiation tissues, 0.448 ± 0.017 (*P* < 0.05). When the Lewis(y) staining intensities of low-differentiation samples were compared with moderate samples or moderate samples were compared with high-differentiation samples, the difference was not significant (*P* > 0.05). These results do not support that the staining intensity of Lewis(y) is correlated with the histologic type of ovarian cancer or with lymph node metastasis (*P* > 0.05) (Table 2).

**Figure 4:**
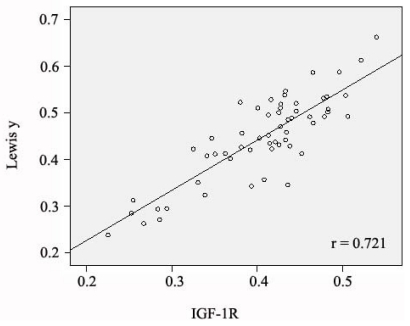
The scatterplot of MOD value of Lewis(y) and IGF-1R in ovarian cancer.

The average optical density of IGF-1R in stage III–IV ovarian cancer was 0.444 ± 0.051, which is significantly higher than that in stage I–II (0.413 ± 0.047) (*P* < 0.05). The average optical density of IGF-1R in low-differentiated ovarian cancer samples was 0.455 ± 0.049, which was significantly higher than the optical densities measured in moderate- (0.413 ± 0.038) (*P* < 0.05) and high-differentiation samples (0.412 ± 0.052) (*P* < 0.05). The difference in optical density between the moderate- and high-differentiation groups was not statistically significant (*P* > 0.05). As with Lewis(y), the expression intensity of IGF-1R was not correlated with the histological type of ovarian cancer or with lymph node metastasis (*P* > 0.05) (Table 2). In most cases, the ovarian cancer tissues that highly expressed Lewis(y) antigen concomitantly expressed high levels of IGF-1R; the expression patterns of these were linearly correlated (*r* = 0.721, *P* < 0.005) (Figure 4).

## 3. Discussion

Cell surface receptors predominantly are populated by glycoproteins, and the sugar chains of which form antenna-like branches to receive information [14–16]. Some hallmarks of malignant tumor cells, such as adhesion, migration, and proliferation, are related to changes in specific sugar chains or residues. Fucosyl residue is a terminal structure of glycan which is involved in the formation of sugar moieties of certain key growth factors. Fucose also plays an important role in growth mechanisms of tumors [7,17]. Lewis(y) antigen is a cancer-associated, difucosylated oligosaccharide that expression in epithelial tissues, and during carcinogenisis, Lewis(y) expression was significantly increased in 60% to 90% patients with a poor prognosis [18]. Yin *et al*. found Lewis(y) has a positive expression rate (75%) and a strong positive expression rate (56%) in ovarian carcinoma respectively. Among 11 types of ovarian cancer cell lines tested, 7 cell lines were found to be Lewis(y) antigen positive [19]. Baldus et al. found that the expression level of Lewis(y) was enhanced with adenoma degeneration and higher histological grade in 44 cases of colorectal adenocarcinoma and 42 cases of colorectal adenomas [20], suggesting that Lewis(y) antigen expression is related to the cell’s degree of malignancy. Our previous work also demonstrated that increasing Lewis(y) expression leads to increased cell proliferation and enhanced adhesion, invasion, metastasis, and drug-resistance [5–7]. In this study, we confirmed our results in tissue samples: Lewis(y) was highly expressed in ovarian cancer tissues, and its positivity rate reached 88.33%, which was significantly higher than the detection rates of marginal cancer tissues (60.00%) (*P* < 0.05) or benign tumors (33.33%) (*P* < 0.01). In addition, the expression intensity of Lewis(y) increased as the level of malignancy increased (*P* < 0.05) and in advanced clinical stages of ovarian cancer (*P* < 0.05). These results indicate positive correlation between the expression of Lewis(y) antigen and the occurrence and development of ovarian cancer.

IGF-1R is a membrane receptor which can be activated by insulin-like growth factor (IGF) and transmit signals into nucleus via many downstream signaling pathways to further act on target genes and involve in regulating the pathogenesis and development of multiple tumors. Numerous studies have confirmed that IGF-1R plays an important role in ovarian cancer progression. An et al. report that expression of IGF-1R was increased in ovarian cancer compared to benign tumors. IGF-1R was found to be higher in tumors with poor prognosis [21]. In this study, IGF-1R expression was consistent with the Lewis(y) antigen results. The positivity rate of IGF-1R in ovarian cancer tissues was 93.33%, significantly higher than in borderline tumor (63.33%), benign tumor (53.33%) (*P* < 0.05), and normal ovarian tissue samples (40%) (*P* < 0.01). The expression intensity of IGF-1R increased with the malignancy level (*P* < 0.05) and was correlated with the operational stage (*P* < 0.05). Analysis of staining intensity in ovarian cancer tissues indicated that Lewis(y) was linearly correlated with IGF-1R (*r* = 0.721, *P* < 0.005). Furthermore, using the double-labeling immunofluorescence method, we found that Lewis(y) and IGF-1R are located in the same position. The evidence suggests that IGF-1R both be involved in the mechanisms underlying development of ovarian cancer and confirms the correlation of Lewis(y) and IGF-1R.

Most epithelial tumor cells overexpress the Lewis(y) antigen, and this may result in Lewis(y)-induced modification of receptor structures on the cell surface. Basu *et al*. reported that epithelial growth factor receptor (EGFR) in Lewis(y) overexpressing tumor cells exhibit surface-exposed Lewis(y) moieties [22]. We detected that Lewis(y) structures were present not only on EGFR but also on CD44 [23,24]. Insulin-like growth factor receptor-1 is initially synthesized as a single-chain proreceptor polypeptide and is processed by glycosylation, proteolytic cleavage, and covalent bonding to assemble into a mature heterotetramer [25]. Despite overexpression and invasion promoting ability of Lewis(y), and IGF-1R being separately reported in various types of human cancer, a direct association between Lewis(y) and IGF-1R has never been described. In this study, we demonstrate that IGF-1R mRNA and protein are elevated in RMG-I-H cells, which was substantially increased with Lewis(y). Exposure to anti-Lewis(y) antibodies could downregulate IGF-1R expression in a time-dependent manner. Lewis(y) was shown to be present in the oligosaccharides of IGF-1R. Notably, the relative content of Lewis(y) structures expressed on IGF-1R was not significantly different between RMG-I and RMG-I-H cells. Our previous results support that Lewis(y) affects cellular biological behaviors (e.g., cell proliferation, apoptosis inhibition) by influencing the activation of signal transduction pathways [26,27]. We speculate that Lewis(y) activated downstream signaling transduction pathways and growth signals are delivered to the nucleus, leading to accelerated gene transcription of IGF-1R in nucleus, and finally promoting the expression of IGF-1R. Then the amplification of IGF-1R gene and overexpression of its protein product closely relate to the poor prognosis in cancer patients.

In summary, transfection of the ovarian cancer cell line RMG-I with α1,2-FT leads to increased Lewis(y) content on surface-expressed IGF-1R proteins, suggesting that the elevated expression level of Lewis(y) and IGF-1R are associated with ovarian cancer. Although the specific mechanisms in this process still need to be elucidated, our results lend insight to a better understanding of the pathogenesis, development, and treatment of ovarian cancer.

## 4. Material and Method

### 4.1. Reagents and Cells

The RMG-I cell line, which was originated from human ovarian clear cell carcinoma, donated by Professor Iwamori Masao of Tokyo University of Japan. α1,2-FT gene transfected RMG-I cell line was established as previously reported [25], named as RMG-I-H. The following reagents were purchased from commercial sources: DMEM and fetal bovine serum (FBS) from Hyclone (Logan, UT, USA); trypsin and ethylenediamine tetraacetic acid (EDTA) from Amresco; Mouse anti-human Lewis(y) mAb (clone A70- C/C8) was purchased from Abcam (England). Rabbit polyclonal anti IGFR1 antibody, HRP-labeled second antibodies and protein G plus-agarose were obtained from Santa Cruz (USA). Goat monoclonal anti-rabbit immunoglobulin G tetramethy lrhodamine isothiocyanate (TRITC) and goat monoclonal anti-mouse immunoglobulin G fluorescein isothiocyanate (FITC) were purchased from Zhongshan Biotechnology (Wuhan, China). The immunohistochemical SP kit was purchased from Mai Xin Company (Fujian, China). Trizol, primers and Reverse Transcription Polymerase Chain Reaction (RT-PCR) reagents came from TaKaRa Company (Dalian, China).

### 4.2. Patients and Tissue Samples

One hundred forty chosen paraffin samples are obtained from the operations done from 2000 to 2009 in the department of gynecology and obstetrics of our hospital. All the tissue sections have a final diagnosis by specialists. There are 60 cases of primary malignant ovarian tumors (including 30 mucous and 30 serous cystadenocarcinomas); 30, borderline ovarian tumor; 30, benign ovarian tumor; and 20, normal ovarian tissues (from the normal ovarian tissue excised in the cervical cancer operations). The mean age of the sepatients is 47.89 years (15–73 years). The age range of the ovarian cancer group is 36 to 73 years; median age is 53.5years. The age range of the border line ovarian tumor group is 22 to 55 years; median age is 35 years. The age ranges of the benign ovarian tumor and normal tissue groups are 15 to 72 and 37 to 52 years, respectively; median ages are 44 and 42 years, respectively. Comparing these groups, there is no statistical significance (*P* > 0.05). According to the pathological grading, the ovarian cancer group contains 21 cases of high differentiation; 21, middle differentiation; and 18, low differentiation. The group includes 39 cases of stages I to II and 21 cases of stages III to VI according to the International Federation of Gynecology and Obstetrics (FIGO) standard; 12 cases of metastases in the cavitas pelvis nodes. All the cases are primary, and the information is complete; chemical treatment is not used in all the patients before operations.

### 4.3. Cell Culture

Cells were cultured in DMEM supplemented with 10% FBS at 37 °C, 5% CO_2_ in humidified air.

### 4.4. Determination of the Expression of IGF-1R mRNAs with Semi-Quantitative RT-PCR

Total RNA was extracted from the transfected and control cells using Trizol reagent. The cDNA was synthesized using Takara RNA PCR Kit and was used as a template for PCR analysis. The primer for IGF-1R was F: 5′-CCCATAGCGTGTTCCCTTTA-3′, R: 5′-CTGGGTGACTCTTGCTCTCC-3′ (fragment size, 874 bp). The primer for β-actin F: 5′-ACAGTCAGCCGCATCTTCTT-3′, R: 5′-GACAAGCTTCCCGTTCTCAG-3′ (fragment size, 259 bp). The cDNA was subjected to denaturation at 95 °C for 5 min, followed by 30 cycles (94 °C, 1 min, 60 °C, 30 s and 72 °C, 30 s) of PCR and incubated at 72 °C for 10 min. After amplification, 10 μL of each reaction mixture was detected by 2% agarose gel electrophoresis.

### 4.5. Analysis of the Proteins of IGF-1R with Western Blotting

Cells were washed twice with ice-cold PBS, scraped in lysis buffer [50 mM Tris-HCl (pH 7.4), 150 mM NaCl, 0.5% NP40, 100 mM NaF, 200 μM Na_3_VO_4_, and 10 μg/mL each aprotinin, leupeptin, PMSF, and pepstatin], and incubated for 30 min at 4 °C while rocking. Lysates were cleared by centrifugation (10 min at 12,000 rpm, 4 °C). For immunoblot analysis, 50 μg of total protein were resolved by SDS-PAGE and transferred to poly(vinylidene difluoride) membranes. Membranes were blocked with TTBS [25 mM Tris-HCl, 150 mM NaCl (pH 7.5), and 0.1% Tween 20] containing 5% nonfat milk and incubated overnight at 4 °C with IGF-1R antibody 1:100 in TBST/1% nonfat milk. Blots were washed in TTBS and incubated with the appropriate horseradish peroxidaselinked IgG, and immunoreactive proteins were visualized with ECL detection system.

### 4.6. Analysis of Lewis(y) Expression on IGF-1R Using Immunoprecipitation and Western Blotting

Washed monolayer cells were lyzed with 200 μL lysis buffer as described above. After protein determination, cell lysate containing 500 μg protein was incubated with 5 μg of IGF-1Rantibody, and incubated at 4 °C for overnight. Protein G plus-agarose was added and the samples were incubated at 4 °C for 3 h for immunoprecipitation.

In brief, immunoprecipitated IGF-1R were subjected to SDS/PAGE, then transferred to a poly(vinylidene difluoride) membrane and treated with 1:1000 diluted anti-Lewis(y) and anti-IGF-1R sera in Tris-buffered saline with 5% fat-free dry milk, followed by 1:1000 HRP-labeled secondary antibody. Finally, the color was developed with enhanced chemiluminescence reagents, and followed by densitometric scanning.

### 4.7. Detection of Lewis(y) and IGF-1R Location on Cells and Tumor Tissue by Double-Labeling Immunofluorescence

RMG-I-H is used to cell climbing slice. Tissue sections are chosen tissues that express a strong positive result in immunohistochemistry using double-labeling immunofluorescence method. The sections are incubated primarily with antibodies against Lewis(y) (1:160) and IGF-1R (1:300) at the same time. Negative control sections are incubated with phosphate buffered saline instead of the primary antibody. The work concentrations of FITC and TRITC are all 1:100. Nuclei were counterstained with DAPI. The empirical procedure is under taken based on kit instruction.

### 4.8. Immunohistochemistry

Histological section of each group of ovarian tissue was 5 μm. The expressions of Lewis(y) and IGF-1R in ovarian carcinoma tissues are analyzed by immunohistochemical streptavidi-peroxidase staining. A colon cancer sample served as positive control for Lewis(y) antigen, and a breast cancer sample was a positive control for IGF-1R. The group with PBS instead of primary antibody was used as a negative control. The work concentrations of primary antibodies against Lewis(y) and IGF-1R are 1:160 and 1:300 respectively. The empirical procedure is under taken based on kit instruction.

### 4.9. Assessment Standard and Statistical Analysis

The presence of brown colored granules on the cell membrane or in the cytoplasm was taken as a positive signal, and was divided by color intensity into not colored, light yellow, brown, tan and is recorded as 0, 1, 2, and 3, respectively. We choose five high-power fields in series from each slice, then score them and take the average percentage of chromatosis cells. A positive cell rate of less than 5% was a score of 0, a positive cell rate of 5~25% was a score of 1, a positive cell rate of 26~50% was a score of 2, positive cell rate of 51~75% was a score of 3, positive cell rate of more than 75% was a score of 4. The final score was determined by multiplying positive cell rate and score values: 0~2 was equal to negative expression (−), 3~4 was equal to weakly positive (+), 5~8 was equal to moderate positive (++), 9~12 was equal to strong positive (+++). The results were read by two independent observers to control for variability.

Microscopic red fluorescence indicated IGF-1R labeled by TRITC, green fluorescence indicated Lewis(y) labeled by FITC. Pictures of the two individual fluorescence channels were superimposed using image analysis software, with a yellow fluorescence indicated co-localization of Lewis(y) antigen and IGF-1R.

The soft ware SPSS version 16.0 (SPSSInc., Chicago, IL, USA) was used for statistical analysis. Data expressed as mean ± SD was applied for statistical analysis. The Student’s t test was applied to compare data between the two groups, and analysis of variance was applied to compare data among multiple groups. The Chi-square (χ^2^) test was applied to analyze the expression of Lewis(y) antigen, IGF-1R and clinicopathological parameters. The correlation between Lewis(y) and IGF-1R expression was tested with the linear regression correlation analysis in ovarian tumor. *P <* 0.05 was considered statistically significant.

## 5. Conclusions

Lewis(y) and IGF-1R are relevant to staging and differentiation of ovarian cancer, suggesting that these two molecules mediate a boosting function for the development of ovarian cancers.

## Figures and Tables

**Table 1:** The expression of Lewis(y) and IGF-1R in different ovarian tissues.

Groups	Cases	Lewis(y)				Positive Cases	Rate (%)	IGF-1R				Positive Cases	Rate (%)
	
		−	**+**	**++**	**+++**			−	**+**	**++**	**+++**		
Malignant group	60	7	15	20	18	53	88.33 *	4	10	23	23	56	93.33 *
Borderline group	30	12	6	11	1	18	60.00†	11	9	7	3	19	63.33‡
Benign group	30	20	6	4	0	10	33.33	14	9	6	1	16	53.33
Normal group	20	20	0	0	0	0	00.00	12	6	2	0	8	40.00

* Compared with borderline and benign group,

† Compared with the benign group;

‡ Compared with benign and normal tissue group.

**Table 2:** Association between Lewis(y) and IGF-1R expression, expression intensity and pathological features in ovarian cancer.

Features		Case	Lewis(y)					IGF-1R				

			Positive Cases	Rate (%)	*P*	MOD	*P*	Positive Cases	Rate (%)	*P*	MOD	*P*
Pathological type	Mucous	30	26	86.67	>0.05	0.463 ± 0.068	>0.05	24	80.00	>0.05	0.418 ± 0.044	>0.05
	Serous	30	27	90.00		0.477 ± 0.016		27	90.00		0.437 ± 0.051	
FIGO stage	I–II	39	33	84.62	>0.05	0.455 ± 0.065	<0.05	33	84.62	>0.05	0.413 ± 0.047	<0.05
	III–IV	21	20	95.24		0.505 ± 0.072		18	85.71		0.444 ± 0.051	
Differentiation level	High	21	17	80.95	>0.05	0.448 ± 0.017	<0.05 *	16	76.19	>0.05	0.412 ± 0.052	<0.05 *
	Middle	21	18	85.71		0.461 ± 0.054	>0.05 †	18	85.71		0.413 ± 0.038	<0.05 †
	Low	18	18	100		0.498 ± 0.084	>0.05 ‡	17	94.44		0.455 ± 0.049	>0.05 ‡
Lymphatic metastasis	No	48	41	85.42	>0.05	0.459 ± 0.078	>0.05	40	83.33	>0.05	0.420 ± 0.054	>0.05
	Yes	12	12	100		0.476 ± 0.057		11	91.67		0.433 ± 0.060	

* Compared the low- with high-differentiation groups;

† Compared the low- with middle-differentiation groups;

‡ Compared the middle- with high-differentiation groups.
